# “Tossing a coin:” defining the excessive use of short-acting beta_2_-agonists in asthma—the views of general practitioners and asthma experts in primary and secondary care

**DOI:** 10.1038/s41533-018-0096-4

**Published:** 2018-07-18

**Authors:** Shauna McKibben, Andy Bush, Mike Thomas, Chris Griffiths

**Affiliations:** 1 Asthma UK Centre for Applied Research, Barts Institute for Population Health Sciences, Barts and The London School of Medicine and Dentistry, Queen Mary University of London, London, UK; 2 Asthma UK Centre for Applied Research, Imperial College and Royal Brompton Hospital, Biomedical Research Unit at the Royal Brompton & Harefield NHS Foundation Trust and Imperial College London, London, UK; 3 Asthma UK Centre for Applied Research, Primary Care and Population Sciences, University of Southampton and NIHR Southampton Biomedical Research Centre, Southampton, UK

## Abstract

The National Review of Asthma Deaths (NRAD) identified high prescribing of short–acting beta_2_-agonists (SABAs) as a key factor in over 40% of deaths. We interviewed asthma experts from both a hospital background (*n* = 5) and a primary care background (*n* = 8), and general practitioners delivering asthma care (*n* = 8), to identify how SABA use is defined and perceived. We identified disparity in how acceptable SABA use is defined, ranging from 0.5 (100 doses/year) to 12 SABA inhalers (2400 doses/year), and complacency in the perception that over-use did not represent a marker for risk of asthma death. Despite current evidence, these findings suggest clinicians of various backgrounds are complacent about excessive SABA use.

## Introduction

Over the past three decades enquiries into asthma deaths in the UK including most recently the National Review of Asthma Deaths “Why asthma still kills,”^1^ (NRAD) have all concluded that the majority of UK asthma deaths are potentially preventable. UK asthma guidelines state that using SABA at least three times a week (300 activations or less than 2 canisters/year) is a marker for potentially poor control and a predictor of future risk of asthma attacks and death.^2^ The NRAD recommended that prescription of more than one SABA per month should trigger an asthma review.^1^ Furthermore, risk of hospital admission has been associated with the prescription of more than three SABAs a year,^3^ whilst morbidity and mortality rise progressively with increasing numbers of SABA dispensed per year.^4,5^ As part of evaluating the use of electronic alerts to highlight excessive SABA prescribing^6^ our aim was to identify how SABA overuse is defined and perceived by general practitioners (GPs) and asthma experts in both community and hospital based practice (Table 1).

**Table 1:** Participant characteristics

Participant characteristics		General practitioners (*n* = 8)	Experts in general practice (*n* = 8)	Experts in hospital care (*n* = 5)
Age	<35	2	0	0
	35–49	5	1	0
	>50	1	7	5
Sex	Male	5	6	1
	Female	3	2	4
Location	UK	8	3	2
	Europe	–	3	2
	International	–	2	1

## Results

We interviewed 21 clinicians; eight general practitioners (GPs) delivering asthma care, eight asthma experts in general practice and five asthma experts in hospital based care. There were wide variations in how excessive SABA use was defined by clinicians with no consensus in regards to how much SABA was excessive. The threshold for acceptable SABA use varied between 0.5 (100 doses) and 12 SABA inhalers (2400 doses) a year. The setting of a SABA use threshold to “tossing a coin” (Expert 3, primary care). Table 2 details the data derived SABA use that clinicians defined acceptable.

**Table 2:** Definitions of acceptable SABA use

SABA threshold	General practitioners (*n* = 8)	Experts in general practice (*n* = 8)	Experts in hospital care (*n* = 5)
<3 times/week or <2 SABA/year	0	3	2
1/month	1	–	2
1/6 months	–	2	–
3–4/year	1	1	–
12/year	2	1	1
Unsure	2	–	–
Did not define	2	1	–

Opinions on the duration of time needed to determine SABA overuse varied between daily and weekly doses and/or monthly inhaler count. One expert commented “GPs in their heart of hearts know that patients shouldn’t have too many SABAs but they really don’t know what too many is” (Expert 1, primary care).

Despite current evidence^1,2,4,5^ some experts questioned the risk of morbidity and mortality associated with high SABA use. Additional factors such as low inhaled corticosteroid use, and markers of asthma attacks such as oral steroid use, hospital admissions and emergency department attendances were deemed necessary to prompt clinical intervention. This suggests a more nuanced approach to identifying and managing those at risk of asthma attacks is necessary.

Asthma guidelines were deemed “stringent” (Expert 10, primary care) whilst guideline recommended SABA use was perceived as “silly” (Expert 3, primary care). One GP commented that guidelines did not reflect the “real world” and that SABA prescribing practice was unlikely to change until someone can “prove” the patient is at increased risk (GP 3).

## Discussion

The challenge of implementing clinical guidelines into practice has been widely documented. However the dismissal of guideline recommended SABA use is surprising, considering the NRAD identified that up to half of the asthma deaths reviewed could have been avoided had asthma guidelines been implemented. There is widespread variation in definitions of excessive SABA use in both research^7–9^ and in practice. Our findings highlight the contrasting perceptions of excessive SABA use among GPs delivering asthma care and those with a specialist interest in asthma.

## Strengths and limitations

The representativeness of the clinicians is a potential limitation due to location and dispersion of roles, therefore it cannot be assumed that data saturation was achieved. However the lack of consensus is itself of real significance and not necessarily a manifestation of inadequate sample size. Further research should address wider national and international perspectives.

## Conclusion

Despite a large body of evidence, there was shocking complacency about SABA over-use and a disregard of current evidence. Unless attitudes can be challenged and changed, it is difficult to see how asthma deaths can be reduced; certainly new guidelines are not the answer.

## Materials and methods

As the study did not involve patients or the use of patient data, it did not require ethical approval by Queen Mary University of London (QMUL; QMERC2061a) and the Health Research Authority. Using convenience and snowball sampling, 14 primary care practices in Tower Hamlets, an ethnically diverse borough in east London, were approached *via* email inviting GPs delivering routine asthma care to participate. Practices were identified with the assistance of the Clinical Effectiveness Group (CEG) at QMUL. Using well-established data sharing networks, the CEG leads and collaborates on research with GP practices in east London. Following co-author discussion, “Asthma Experts” in both community and hospital practice were identified using convenience sampling and invited via email to participate. Asthma Experts were defined as primary or secondary care clinicians who have contributed specifically to asthma care at a national or international level. Semi-structured interviews were carried out in-person (GPs) or via telephone (asthma experts) until no new responses were elicited. The topic guide excluded questions on pre-exercise SABA use (see supplementary material). Written informed consent was obtained from GPs and verbal consent obtained from asthma experts prior to interview. Interviews were audio recorded, transcribed anonymously and analyzed thematically using the Framework Method.^10^ The coding framework underwent six revisions with the assistance of an independent coder.

### Data availability

Authors confirm that all relevant data are included in the paper.

## Electronic supplementary material

Interview guide (SI)

## Electronic supplementary material

**Supplementary information** accompanies the paper on the *npj Primary Care Respiratory Medicine* website (10.1038/s41533-018-0096-4).
