# Author Correction: “Tossing a coin:” defining the excessive use of short-acting beta2-agonists in asthma—the views of general practitioners and asthma experts in primary and secondary care

**DOI:** 10.1038/s41533-018-0114-6

**Published:** 2019-01-14

**Authors:** Shauna McKibben, Andy Bush, Mike Thomas, Chris Griffiths

**Affiliations:** 1 Asthma UK Centre for Applied Research, Barts Institute for Population Health Sciences, Barts and The London School of Medicine and Dentistry, Queen Mary University of London, London, UK; 2 Asthma UK Centre for Applied Research, Imperial College and Royal Brompton Hospital, Biomedical Research Unit at the Royal Brompton & Harefield NHS Foundation Trust and Imperial College London, London, UK; 3 Asthma UK Centre for Applied Research, Primary Care and Population Sciences, University of Southampton and NIHR Southampton Biomedical Research Centre, Southampton, UK

**Correction to:** *npj Primary Care Respiratory Medicine* 10.1038/s41533-018-0096-4; Published online 18 July 2018

In the PDF and HTML versions of this Brief Communication a couple of words are not shown in a sentence in the penultimate sentence of the first paragraph of the Results, changing the meaning. This sentence should have been “The setting of a SABA use threshold was likened to “tossing a coin” (Expert 3, primary care).”

