# Supplementary material for: “Tossing a coin:” defining the excessive use of short-acting beta2-agonists in asthma—the views of general practitioners and asthma experts in primary and secondary care
Source: NPJ Prim Care Respir Med. 2018 Jul 18;28:26. doi: 10.1038/s41533-018-0096-4 (PMC6052065; doi:10.1038/s41533-018-0096-4)
Supplement: Supplementary file 1 — Interview guide (SI) [file 41533_2018_96_MOESM1_ESM.pdf]

Supplementary material

Topic guide for interviews	
Identifying and managing high SABA use	How would you define excessive SABA use? Excluding pre-exercise use.<br>PROMPT: volume and duration, evidence e.g. National Review of Asthma Deaths, BTS/GINA guidelines, other research
	How do you identify patients prescribed high numbers of SABA inhalers?<br>PROMPT: methods: computerised/manual and context,<br>task: consultation/repeat prescribing, workflow
	What happens if high SABA prescribing is identified?<br>PROMPT: type of action, challenges to action, who is involved
	Who is involved in identifying and managing high SABA use?<br>PROMPT: the role of reception, pharmacy, nurse, patient
Alerts to identify high SABA use	What are your thoughts on an alert to identify patients being prescribed excessive SABA?<br>PROMPT: current EMIS medicines management alert
	How do you/would you use this alert?<br>PROMPT: In what context, how do you respond
	How could an alert be improved?
Additional questions for primary and secondary experts	What are your thoughts on recommendations for electronic surveillance of SABA prescribing in primary care practices?<br>PROMPT: National Review of Asthma Deaths, current EMIS medicines management alert
	How do you feel this could best be done?
	What do you view as challenges to such a system? How could these challenges be overcome?
	Who should such a system involve?
	How should the success of such a system be measured?
